# In this issue

**Published:** 2022-07

**Eye lens opacities and cataracts among physicians and healthcare workers occupationally exposed to radiation.** *A systematic review and meta-analysis*

Alhasan & Aalam evaluate the risk of developing eye lens opacities and cataracts among physicians and healthcare workers occupationally exposed to radiation. Healthcare workers exposed to radiation have a significantly greater risk of posterior subcapsular cataracts (PSCs), cataracts, and any lens opacities than those of the non-exposed participants. Subgroup analysis reveal that nurses have the highest risk for PSCs, followed by interventional cardiologists. They concluded that the risk of posterior subcapsular opacities and cataracts was significantly higher in healthcare workers with occupational radiation exposure than in non-exposed workers, highlighting the necessity to enhance and promote the wearing of protective measures with high safety levels.

**Figure:**
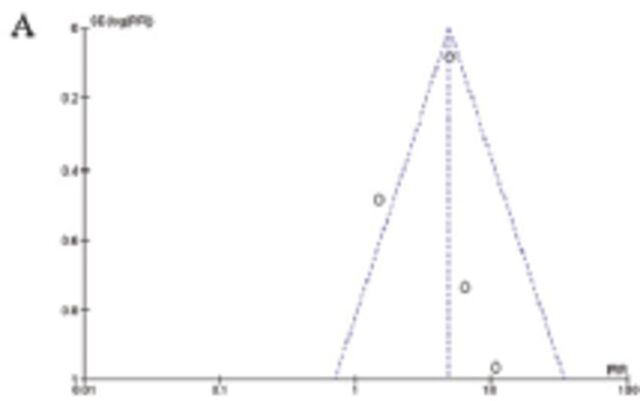
Funnel plots demonstrating no proof of publication bias in the included articles.

***see page 665***

