# Supplementary material for: In this issue
Source: Saudi Med J. 2022 Jul;43(7):663. (PMC9749695)
Supplement: Related texts

# Abstract: Original Articles

**Comparison of bone mineral density and Fracture Risk Assessment Tool in Saudi women with and without type 2 diabetes mellitus.** *A cross-sectional study*

Alfadhli et al compare the bone mineral density and the fracture risks in Saudi women with and without type 2 diabetes mellitus (T2DM). The 10-year probabilities of major osteoporotic fracture (MOF) and hip fracture (HF) are calculated using the Abu Dhabi Fracture Risk Assessment Tool (FRAX) with and without adjustment for T2DM. There are no significant differences in mean age, menopausal status, height, weight, and body mass index between T2DM and non-diabetic women. Bone mineral density values and the unadjusted FRAX scores are comparable between the 2 groups. However, after adjusting FRAX for T2DM, the FRAX for MOF and HF becomes significantly higher in T2DM women. They concluded that in Saudi women with T2DM, unadjusted FRAX underestimated the risk of MOF and HF. Type 2 diabetes mellitus should be included as one of the clinical risk factors for fracture in future versions of the FRAX score.

**Figure:** The fracture risk assessment score for major osteoporotic fracture in type 2 diabetes women versus non-diabetes with and without rheumatoid arthritis adjustment.

***see page 700***

**D-dimer as a predictive and prognostic marker among COVID-19 patients**

Elkhalifa examines D-dimer, coagulation profile, and platelet count among patients hospitalized with coronavirus disease-19 (COVID-19) and compare them to findings from non-COVID-19 subjects. The participants in this retrospective hospital-based observational study design included 112 confirmed diagnosed with COVID-19 and another 112 non-COVID-19 subjects as a comparative group. Pneumonia related to COVID-19 is classified as critical, severe, moderate, mild, and asymptomatic whereas thrombocytopenia is marked when the platelet count is <150.00×109/L. The D-dimer and activated partial thromboplastin clotting time mean values are significantly elevated. The international normalized ratio and platelet count mean values confirms a significant decrease. Thrombocytopenia is found 9 times in COVID-19 higher than in the non-COVID-19. D-dimer and prothrombin time mean values increase significantly among the COVID-19 patients with all patterns of symptoms on admission. He concluded that D-dimer mean values increased significantly in deceased COVID-19 and in hospitalized intensive care unit (ICU) wards patients, indicating a potential predictive and prognostic severity marker, particularly among COVID-19 patients in the ICU.

**Figure:** Receiver operating characteristic curve analysis of coronavirus disease-19 patients D-dimer.

***see page 723***

# Abstract: Case Report

**Hidradenitis supparativa complicated by penoscrotal lymphedema and renal amyloidosis**

Bin Rubaian et al report a case of a 42-year-old Indian gentleman who has hidradenitis suppurativa (HS) for 9 years presenting with 2 rare complications: penoscrotal lymphedema and end-stage renal disease secondary to renal amyloidosis. The patient is treated with prednisolone and adalimumab to treat both his HS and renal amyloidosis, and is referred to general surgery to manage his genital lymphedema. They recommended following adult patients with moderate-to-severe HS and clinical duration of greater than 3 years and screening for amyloidosis before they reach end-stage organ disease, similar to what happened to their patient with end-stage renal disease.

**Figure:** A glomerulus shows abundant mesangial as well as capillary wall deposits of amorphous eosinophilic material (H&E - x400).

***see page 723***
